# Large and Anisotropic Linear Magnetoresistance in Single Crystals of Black Phosphorus Arising From Mobility Fluctuations

**DOI:** 10.1038/srep23807

**Published:** 2016-03-31

**Authors:** Zhipeng Hou, Bingchao Yang, Yue Wang, Bei Ding, Xiaoming Zhang, Yuan Yao, Enke Liu, Xuekui Xi, Guangheng Wu, Zhongming Zeng, Zhongyuan Liu, Wenhong Wang

**Affiliations:** 1 Beijing National Laboratory for Condensed Matter Physics, Institute of Physics, Chinese Academy of Sciences, Beijing 100190, China; 2 State Key Laboratory of Metastable Materials Science and Technology, Yanshan University, Qinghuangdao 066004, China; 3 Key Laboratory of Nanodevices and Applications,Suzhou Institute of Nano-tech and Nano-bionics, Chinese Academy of Sciences, Ruoshui Road 398, Suzhou 215123, China

## Abstract

Black Phosphorus (BP) is presently attracting immense research interest on the global level due to its high mobility and suitable band gap for potential application in optoelectronics and flexible devices. It was theoretically predicted that BP has a large direction-dependent electrical and magnetotransport anisotropy. Investigations on magnetotransport of BP may therefore provide a new platform for studying the nature of electron transport in layered materials. However, to the best of our knowledge, magnetotransport studies, especially the anisotropic magnetoresistance (MR) effect in layered BP, are rarely reported. Here, we report a large linear MR up to 510% at a magnetic field of 7 Tesla in single crystals of BP. Analysis of the temperature and angle dependence of MR revealed that the large linear MR in our sample originates from mobility fluctuations. Furthermore, we reveal that the large linear MR of layered BP in fact follows a three-dimensional behavior rather than a two-dimensional one. Our results have implications to both the fundamental understanding and magnetoresistive device applications of BP.

Black phosphorus (BP), an emerging layered two-dimensional (2D) semiconductor, is presently attracting immense research interest on the global level due to its high mobility and suitable band gap for potential applications in novel electronic and optoelectronic devices^1^,^2^,^3^,^4^,^5^. Unlike other well-studied 2D materials, such as semimetallic graphene^6^ with zero band gap and MoS_2_^7^ with a direct band gap of ~1.5 eV only in its monolayer form, BP has a tunable thickness-dependent direct band gap varying from ~0.3 eV (bulk) to >1.4 eV (monolayer)^4^,^5^. This prominent feature benefits layered BP in optoelectronic applications such as phototransistors, *p-n* diodes and solar cells^2^,^8^,^9^,^10^,^11^. So far, BP has been shown to have some remarkable properties, such as high mobility^1^,^2^, semiconductor-metal transition^12^ as well as superconductivity^13^ under high pressure. Additionally, as shown in Fig. 1(a), owning to the puckered honeycomb lattice in each P atom layer, much work has been involved to explore the anisotropic thermal transport properties in bulk BP^14^,^15^,^16^, indicating that BP could be also used as a novel thermoelectric material in which the anisotropic properties might be used. Similar as the anisotropic thermal conductivity, it was theoretically predicted that BP has a large direction-dependent photonic^17^,^18^ and magneto-transport anisotropy^1^. Investigations on magneto-transport properties of BP may therefore provide a new platform for study the nature of electron transport in layered materials. Importantly, the pressure-induced electronic transition and colossal magnetoresistance (MR) have been recently reported in bulk BP^19^, suggesting that BP can be used for potential application in future magneto-electronic devices. However, magneto-transport studies, especially the anisotropic MR effect in layered BP, are rarely reported. To the best of our knowledge, the only anisotropy conductivity measurement of BP was conducted in 1983 by Akahama *et al*.^20^, which neither observed the large linear magnetoresistance (LMR) nor anisotropy in bulk BP.

In the present work, we report a large and anisotropic LMR effect up to 510% at a magnetic field of 7 T in single crystals of BP when the electronic current is applied within the cleaved *ac*-plane (current flows along the *c*-axis) and the magnetic field is applied perpendicular to the current direction, along the *b*-axis. Analysis of the temperature dependence of transport properties reveals that the large LMR in our samples originates from mobility fluctuations. We further demonstrate that the LMR follows a three-dimensional (3D) behavior with a small mass anisotropy.

## Results and Discussion

**Figure 1:**
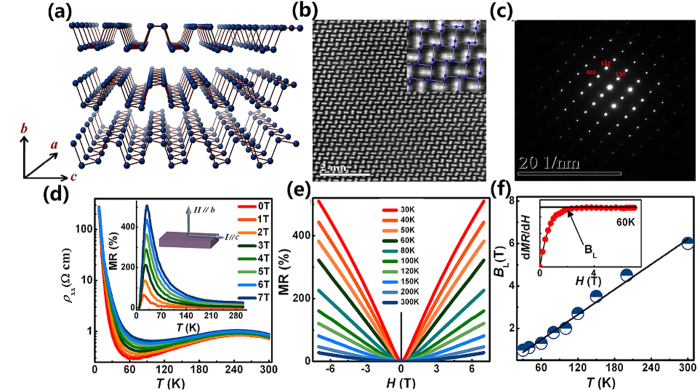
Crystal structure and large LMR of BP. (**a**) Typical perspective side view of the crystal structure of bulk BP. (**b**) High-resolution HADDF image of a BP flake, which shows the atom chains clearly. (**c**) Selected area electron diffraction pattern taken from the area shown in (**c**). (**d**) The temperature dependence of resistivity *ρ*_xx_ in various magnetic fields with the current parallel to the *c* axis and the magnetic field perpendicular to the current along the *b*-axis at the temperature range from 300 K to 10 K. (**e**) Normalized LMR as a function of magnetic field *H* at a series of temperatures. Here MR = [*ρ* (*H*) −*ρ* (0)]/*ρ* (0)]× 100%, where *ρ* (*H*) and *ρ* (0) are the resistivity with and without the magnetic field *H*, respectively. (**f**) The crossover field *B*_L_ as a function of temperature. Inset shows the first derivative of MR with external magnetic field at 60 K, the intersection of lines indicates a turning point between parabolic and LMR.

Single crystals of BP that were prepared by using the high-pressure synthesis technique described in the Methods section. The samples with a typical size of about 3.0 × 1.0 × 0.1 mm^3^ were cleaved at room temperature in an argon-filled glove box resulting in the well-developed and shining (0*l*0) surfaces as shwon in Supplementary Fig. S1. The X-ray diffraction (XRD) pattern and Raman spectrum of ground BP powders are consistent with the reported ones of bulk BP crystals^21^,^22^, indicating the good quality of our prepared bulk BP. Exfoliated BP flakes were analysed using a double Cs-corrected high-resolution transmission electron microscipe along the [110] zoen axis, as shown in Fig. 1b. Each flake has identical lattice structure with its corresponding selected area electron diffraction pattern shown in the Fig. 1c, confirming that the flake is a single crystal. The electrical contacts were arranged in a conventional four-probe configuration. To protect the samples from oxidation, a varnish film was made on the sample surface. The magnetotransport measurements were preformed in a Quantum Design PPMS-9 in a maximum magentic field of 10 T at the temperature range of 300 K–10 K. A sketch of the experimental configurations in this study is shown inset of Fig. 1d where the current is applied parallel to the *ac*-plane (current flows along the *c*-axis), with the magnetic field applied perpendicular to the *ac*-plane (i.e., H//*b*). More than five samples are prepared with different thickness and size. In the main text, we just present the transport properties of the sample with the maximum MR. For comparison, the detail data of another typical sample were shown in the Supplementary Information. In Fig. 1d, we show the temperature dependence of resistivity *ρ*_xx_ in various magnetic fields at the temperature range from 300 K to 10 K. In zero field, an usual behavior of intrinsic semiconductor is observed at the temperature range of 300 K–250 K, and we can obtain a resistivity as large as 0.72 Ω cm at 300 K which is in the same order as that of the previous reported bulk BP^19^,^20^. With decreasing temperature from 250 K, *ρ*_xx_ firstly decreases in a metallic manner reaching a minimum value at 60 K. This tansition point can be attributed to the thermal activation of impurity donors which is quite likely to be disorders in our BP samples. With the further decrease of temperature, as the donors freeze out, *ρ*_xx_ exhibits an exponential increase again with a smaller band gap energy *E*_g_ = 14.6 mev (see Supplementary Fig. S1). When we applied an external magnetic field, the temperature-dependent behavior of resistivity is not changed significantly, however its value increases sharply with the increase of magnetic field especially at the temperature range of 180–30 K. In the inset of Fig. 1d, we plot the deduced MR in various magnetic fields as a function of temperature. The MR is defined as [*ρ*(H)-*ρ*(0)]/*ρ*(0)]×100%, where *ρ*(*H*) and *ρ*(0) are the resistivity at field *H* and zero, respectively. At 1 T, MR firstly increases with the decrease of temperature and reaches a maximum of 51% at 20 K, and then decreases drastically with the further decrease of temperature. With the increase of magnetic field, the maximum MR together with its corresponding temperature increases and a large MR of 510% was observed at 30 K in 7 T. Also, the field sensitivity of MR was established to exceed 70%/T, which makes layered BP potential applications in the future magnetoresietance devices.

To reveal the relationship between the MR and external magnetic field, in Fig. 1e, we present an overview of the magnetic field dependence of the MR at a series of temperatures. At 300 K, MR shows a quadratic growth below a threshold field *B*_L_ (6 T at 300 K as shown in Fig. 1f) and then transforms into a linearly rising behavior with the increase of magnetic field without sign of saturation. With the decrease of temperature, the value of *B*_L_ decreases, especially below 100 K, the crossover field falls down to 1 T with a tendency towards a slightly sublinear dependence in the high magnetic region at low temperatures (see Supplementary Fig. S2 and Fig. S3). Strikingly, only a slight difference in the magnitude of MR was observed in different samples (see Supplementary Fig. S4 and Fig. S5), which further supports the high-quality of BP crystals. Remarkably, a pronounced cusp of MR was founded in the low-field region at temperatrues below 20 K (see Supplementary Fig. S3 and Fig. S5). This phenomenon is reminiscent of the weak antilocalization (WAL) effect that has widely been reported in materials with strong spin-orbital coupling (SOC)^23^,^24^ or topological insulators^25^,^26^. It should be pointed that similar results have been observed in the graphen^27^,^28^, in which the carriers have a Berry phase of π due to its chiral character. However, further investigations are required to verify the origin of the WAL in layered BP.

**Figure 2:**
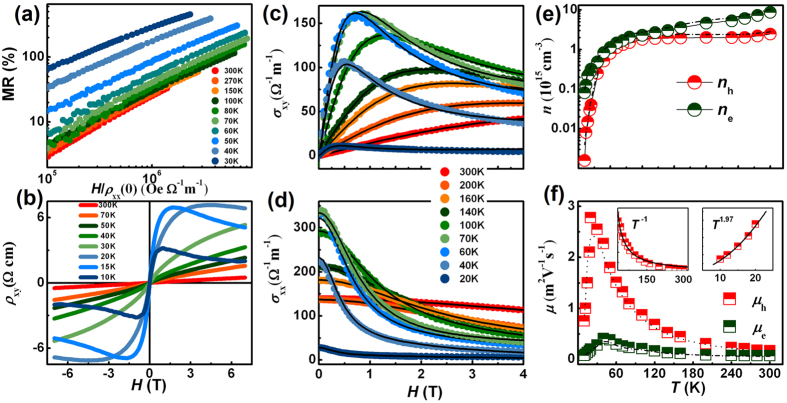
Transport properties of BP. (**a**) The Kohler plot: the MR as a function of *H*/*ρ*_xx_(0). The MR data above 100 K nearly collapses onto a single universal curve scaled linearly with *H* and obeys Kohler’s rule, but completely deviates from the Kohler’s rule below 100 K. (**b**) The magnetic field dependence of Hall resistivity *ρ*_xy_ at different temperatures. (**c**) Hall conductivity *σ*_xy_ and (**d**) longitudinal conductivity *σ*_xx_ at selected temperatures. The solid curves are the results of calculations using the two-carrier model. (**e**) Temperature dependence of hole concentration *n*_h_ and electron concentration *n*_e_ estimated from *σ*_xx_. The dotted lines show the hole concentration and electron concentration estimated from *σ*_xy_. (**f**) Temperature dependence of hole mobility *μ*_h_ and electron mobility *μ*_e_ estimated from *σ*_xx_. The dotted lines show the hole mobility and electron mobility estimated from *σ*_xy_. The left inset: the hole mobility above 30 K is fitted by *T*^−1^. The right inset: the hole mobility below 30 K is fitted by *T*^1.97^.

Figure 2a presents the so-called Kohler plot^29^, in which the MR ratio is plotted as a function of *H*/*ρ*_xx_(0). Above 100 K, MR data nearly collapse onto a single universal curve scaled linearly with *H* though a small deviation from Kohler’s rule is observed, indicating a single relevant scattering process is dominant in bulk BP. When the temperature decreases below 100 K, complete deviation from Kohler’s rule is found, suggesting that more than one type of carrier dominate the electronic transport properties. To gain a further insight into the carrier transport in BP crystals, we performed the Hall effect measurements with the current along the *c*-axis (The anisotropy is small with the current along the *a*-axis, as shown in Supplementary Fig. S6) and the magnetic field along the *b*-axis. Fig. 2b shows the corresponding magnetic field dependence of Hall resistivity *ρ*_xy_ at selected temperatures. At the whole temperature range, the positive slope indicates the predominance of hole carriers. However, nonlinear field dependence of *ρ*_xy_ is observed below 100 K in the high field region, which concides with the Kohler’s rule and signals that more than one carrier domain the transport properties. Moreover, it is of interest to notice that the slope of *ρ*_xy_ changes from positive into negative with the increase of magnetic field below 20 K. This behavior has been observed in the bulk BP under pressure^19^, graphene^30^, NbSb_2_^31^, and LuPtBi semimetal^32^, which is supposed to be the change of band structure induced by magnetic field or pressure. Considering the positive slope at low field and its highly nonlinear *H* dependence, it is reasonable to assume that the bulk BP has two types of carrier with different scattering time, one is the high-mobility hole carrier and the other is the low-mobility electron carrier.

Based on the above scenarios, the conductivity tensors are analyzed by the two-carrier model^33^ to determine the mobility and concentration of hole and electron carriers, respectively, as described in Methods. Using the fitting results both for Hall conductivity *σ*_xy_ (Fig. 2c) and longitudinal conductivity *σ*_xx_ (Fig. 2d), we can independently obtain the Hall and longitudinal mobilities and concentrations for hole and electron carriers, respectively. We found that, as shown in Fig. 2e,f, both the Hall and longitudinal mobilities and carrier concentrations agree well with each other in the whole temperature range, indicating the validity of the two-carrier model in our fitting procedure. In addition, as shown in Fig. 2e, at temperatures above 250 K, the hole concentration firstly decreases with the decrease of temperature which is consistent with the usual behavior of intrinsic semiconductor. In the temperature range of 250 K–50 K, the impurities are thermal activated, which leads to a temperature-independent behavior. At temperatures below 50 K, the concentration decreases drastically and an extremely low carrier concentration of 1.58 × 10^12^ cm^−3^ was obtained at 10 K. As shown in Fig. 2f, the hole mobility of BP starts with a *T*^−1^ behavior up to 30 K, and then follows a negligible temperature variation of *T*^2^ when the temperature decreases below 30 K. The behavior of temperature-dependent mobility in our bulk BP agrees well with the previously reported few-layers BP^1^,^2^,^3^,^4^, however the value (in the range of 28000–1700 cm^2^V^−1^s^−1^) is nearly two orders of magnitude larger than the later one. Moreover, based on the power law, we can deduce that the lattice vibration scattering dominates the temperature-dependent mobility at the temperature range of 300 K–30 K, whereas the ionized impurity scattering dominates the low-temperature mobility.

**Figure 3:**
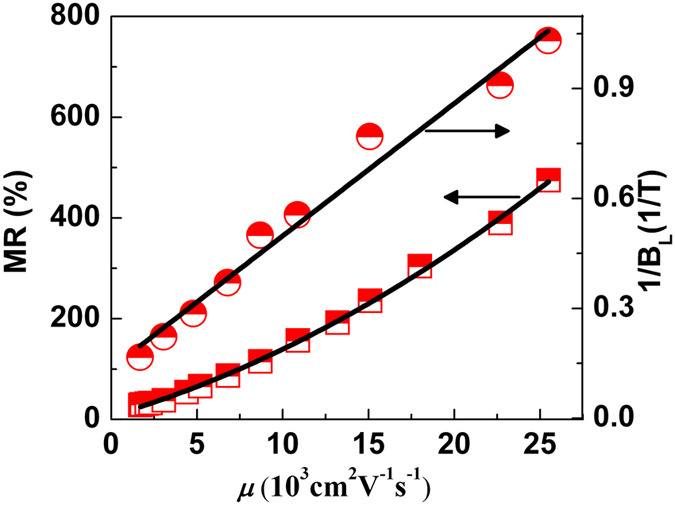
PL model for the origin of LMR in bulk BP. The data of mobility vs. MR in 7 T and reciprocal of the crossover field 1/*B*_L_.

Next we will discuss the origin of the large LMR in BP crystals. It is well known that the positive LMR can arise from either quantum^34^ or classical effects^35^. On the one hand, we did not observe the Shubnikov-de Haas (SdH) oscillations to support the presence of quantum MR even at low temperatures and high fields (see Supplementary Fig. S7), and we can thus rule out the quantum origin of the LMR in BP. On the other hand, as we will show below that the presence of LMR can be well described by the classical disorder model, where the LMR is expected to be governed by carrier mobility. The classical LMR has been observed in several material systems, including semimetals^36^,^37^,^38^,^39^, narrow band-gap semiconductors^40^, multi-layer graphene^41^, and topological insulators^42^,^43^. The core of this model is the inhomogeneities produce the large spatial fluctuations in the conductor tensor, which leads to the electronic cycloidal trajectories around low-mobility islands and induces the LMR. Based on this model, the linear crossover field *B*_L_ (the amplitude of MR) is inverse proportion (proportion) of carrier mobility. In Fig. 3, we plot the values of MR in 7 T at the temperature range 300 K–30 K where exhibits both the LMR and the crossover field 1/*B*_L_ as a function of mobility, it is obviously found that both the MR and 1/*B*_L_ increase correspondingly with the increase of carrier mobility. We can therefore conclude that the LMR of layered BP crystals is a classical effect and originates from mobility fluctuations due to multiple-electron scattering of high-mobility carriers by low-mobility islands in bulk BP.

**Figure 4:**
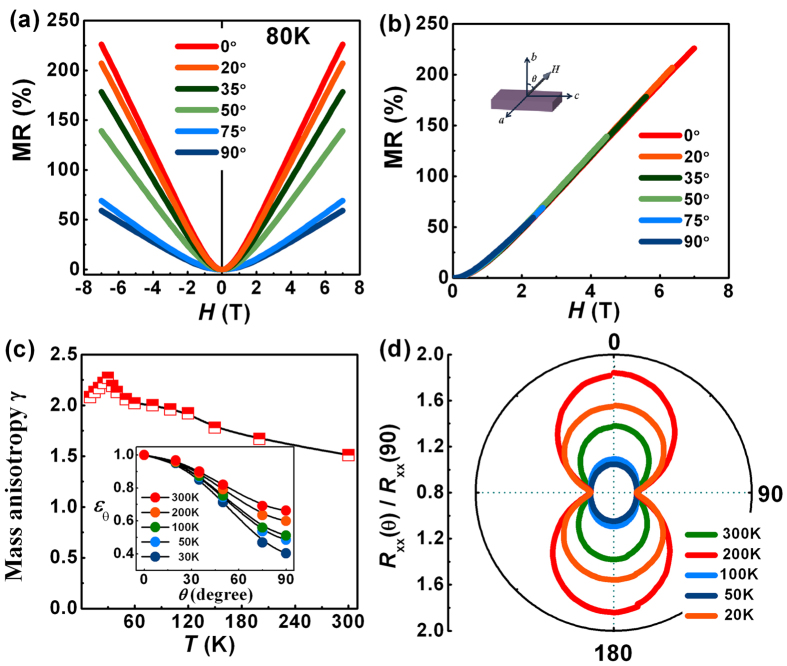
Scaling behavior of the field dependence of MR and the temperature dependence of mass anisotropy *γ*. (**a**) The field dependence of the MR obtained in various magnetic field orientations at 80 K. (**b**) Data in (**a**) plotted with *H* scaled by a factor *ε*_*θ*_. The inset shows the schematic of measurement where *θ* suggests the angle between the magnetic field and the normal direction of *ac*-plane. *θ* = 90° means the magnetic field is parallel to ac-plane. (**c**) The temperature dependence of *γ*. Inset: angle dependence of *ε*_*θ*_ at different temperatures. (**d**) Polar plots of the angle-dependent *R*_xx_(θ)/*R*_xx_(90°) at different temperatures in a magnetic field of 5 T.

Let us turn on our attention to the anisotropy of MR in BP crystals. Figure 4a shows the magnetic field dependence of MR for sample I at 80 K at various angle *θ* (see the inset of Fig. 4b for the definition of *θ*). Our data reveals that the amplitude of MR is anisotropic with larger LMR for an external magnetic field closer to the *b*-axis (*θ* = 0*°*). For a 2D system, the LMR at *θ* = 90*°* should decrease to quite a small value due to the decrease of contribution of magnetic field and MR vs. the field scaling factor *ε*_*θ*_ = *H* cos *θ* curves also should overlap onto one curve. However, as shown in Fig. 4a, the MR of BP at *θ* = 90*°* still keeps a large value and the LMR vs. *H* curves deviate from each other (see Supplementary Fig. S8), proving that the 3D bulk transport may contribute the anisotropy of LMR in BP crystals. In order to obtain the contribution of anisotropy of band structure, the factor of anisotropy of 3D band structure was added into the scaling factor *ε*_*θ*_, and *ε*_*θ*_ can be expressed as follow:


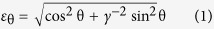


where *γ* reflects the ratio of the effective masses of electrons moving in direction of *θ* =* *0*°* and 90*°.* As mentioned above, the LMR in the bulk BP is proportion of the mobility *μ*, in the semi-classical model, the resistance *R* is closely related to the mobility by the relation *R* = 1/ne*μ*, where the mobility can be expressed as *μ* = eτ/m* with *τ* being the relaxation time, m* the effective mass, and *e* the electron charge. Therefore, the anisotropy of effective mass is expected to play an essential role in the anisotropy of LMR. As shown in Fig. 4b, the MR curves at 80 K at various *θ* can be collapsed onto one single curve with the field scaling factor *ε*_*θ*_ and thus we can determine the value of *γ* to be 2.1, which is much smaller that of (~12.1) the well-known 2D graphite^44^. However, this value is similar to that of (~2 at 100 K) the layered WTe_2_ showing a 3D Fermi surface of moderate anisotropy^45^. The small anisotropy of BP may arise from the unique puckered atomic structure in the out-of-plane direction, which is similar to case of layered WTe_2_ that exhibits the distortion of the tellurium layers.

Figure 4c shows the temperature dependence of *γ*, which is deduced from the anisotropic MR data. It can be seen that the values fall within the range of 1.5–2.3, and we can notice that the value of *γ* firstly increases correspondingly with the decrease of temperature reaching a maximum value at 30 K and then decreases with the further decrease of temperature, which coincides well with the change tendency of mobility and MR. In Fig. 4d, the angle dependence of MR at various temperatures is shown. It is clearly evident that the anisotropy reaches the largest at 30 K, which reflects the change tendency of band structure and agrees well with the temperature dependence of *γ.* We should point out that, the recent angle-resolved photoemission spectroscopy experiments have revealed that the band width along the out-of-plane direction becomes smaller at temperature below 30 K, which indicated the charge carriers of bulk BP are more localized in the 2D plane at low temperatures^46^. Here, we suppose that the temperature induces the dispersion of band transforms gradually from quadratic gradually towards the linear which exhibits a larger anisotropy of effective mass and therefore a higher mobility and larger MR.

In conclusion, we have observed for the first time a large and anisotropic LMR in single crystals of BP. The large LMR is up to 510% at a magnetic field of 7T when the electronic current is applied along the *c*-axis within the cleaved *ac*-plane and the magnetic field is applied perpendicular to the current direction, along the *b* axis. Detailed transport measurement revealed that the large LMR is a classical effect and originates from mobility fluctuations due to multiple-electron scattering of high-mobility carriers by low-mobility islands in bulk BP. By using angular-dependent MR measurements, we further demonstrated that large LMR of layered BP in fact follows a three-dimensional behavior rather than a two-dimensional one. Our results have implications to both the fundamental understanding and magnetoresistive device applications of BP.

## Methods

### Sample preparation

Bulk black phosphorous (BP) was prepared from red phosphorous at high temperature of 800 °C and high pressure of 2 GPa for 10 min. The prepared bulk BP was cleaved or ground to powder in a glove box filled with Ar gas for the subsequent use.

### Materials Characterization

The ground BP powder was characterized by the SEM, XRD and Raman measurements. The SEM image was taken by a scanning electron microscopy (S-4800, Hitachi, Japan). The XRD patterns were collected on SmartLab with Cu-Kα radiation (λ = 1.5406 Å, Rigaku, Japan). The Raman measurement was carried out on a Renishaw inVia micro-Raman spectroscopy with a laser radiation of 514 nm. The dispersion BP sheets were dropped onto holey carbon support film with 200 mesh copper grids TEM and SAED. The TEM images and SAEDs of the BP sheets were obtained in a transmission electron microscopy (JEM-2010, JEOL, Japan).

### Transport measurements

The cleaved samples with a typical size of about 3.0 × 1.0 × 0.1 mm^3^ were used for transport measurements. The standard four-probe Hall (*R*_xy_) and resistive (*R*_xx_) measurements were carried out in a Quantum Design PPMS-9 using a constant current mode. For all measurements except for the angular dependence of the transverse magnetoresistance (MR), the magnetic field *H* was applied perpendicular to the cleaved ac plane of the crystal. The *R*_xx_(H) and *R*_xy_(H) were measured by sweeping *H* between ±7 T at fixed temperatures. Angular dependence of the transverse MR was measured for different orientations, where *θ* being the angle between *H* and the normal to the ac plane. Electrical contacts were prepared by platinum wires and a silver paste.

### Two-carrier model analyses

The conductor tensors of BP crystal are analyzed by a two–carrier model in which one carrier is of high mobility and the other is of low mobility. The longitudinal conductivity *σ*_xx_ and Hall conductivity *σ*_xy_ can be described as:


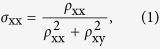



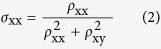


Thus, in field regime (0–5T), the carrier concentrations and mobilities can be independently extracted by fitting *σ*_xx_ and *σ*_xy_ with the two following Equations


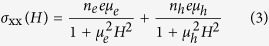



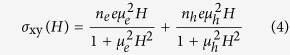


The *n*_e_ (*n*_h_) and *μ*_e_ (*μ*_h_) indicate the carrier concentrations and carrier mobilities of electron (hole), respectively. The fitting parameters are dependent on temperature *T*, but independent on external magnetic field *H*.

## Additional Information

**How to cite this article**: Hou, Z. *et al*. Large and Anisotropic Linear Magnetoresistance in Single Crystals of Black Phosphorus Arising From Mobility Fluctuations. *Sci. Rep.* **6**, 23807; doi: 10.1038/srep23807 (2016).

## Supplementary Material

Supplementary Information

## References

[b1] Qiao J. S., Kong X. H., Hu Z. X., Yang F. & Ji W. High-mobility transport anisotropy and linear dichroism in few-layer black phosphorus. Nat. Commun. 5, 4475 (2014). 25042376 10.1038/ncomms5475 PMC4109013

[b2] Xia F. N., Wang H. & Jia Y. C. Rediscovering black phosphorus as an anisotropic layered material for optoelectronics and electronics. Nat. Commun. 5, 4458 (2014). 25041752 10.1038/ncomms5458

[b3] Kim J. M. . Observation of tunable band gap and anisotropic Dirac semimetal state in black phosphorus. Science 349, 723 (2015). 26273052 10.1126/science.aaa6486

[b4] Tran V., Soklaski R., Liang Y. F. & Yang L. Layer-controlled band gap and anisotropic excitons in few-layer black phosphorus. Phys. Rev. B. 89, 235319 (2004).

[b5] Liang L. . Electronic bandgap and edge reconstruction in phosphorene materials. Nano lett. 14, 6400 (2014). 25343376 10.1021/nl502892t

[b6] Novoselov K. S. . Two-dimensional gas of massless Dirac fermions in graphene. Nature 438, 197 (2005). 16281030 10.1038/nature04233

[b7] Wang Q. H., Kalantar-Zadeh K., Kis A., Coleman J. N. & Strano M. S. Electronics and optoelectronics of two-dimensional transition metal dichalcogenides. Nat. Nanotechnol. 7, 699 (2012). 23132225 10.1038/nnano.2012.193

[b8] Buscerma M. . Fast and broadband photoresponse of few-layer black phosphorus field-effect transistors. Nano Lett. 14, 3347 (2014). 24821381 10.1021/nl5008085

[b9] Deng Y. . Black phosphorus-monolayer MoS_2_ van der Waals heterojunction p-n diode. ACS Nano 8, 8292 (2014). 25019534 10.1021/nn5027388

[b10] Engel M., Steiner M. & Avouris P. Black phosphorus photodetector for multispectral, high-resolution imaging. Nano lett. 14, 6414 (2004). 25299161 10.1021/nl502928y

[b11] Dai J. & Zeng X. C. Bilayer phosphorene: effect of stacking order on bandgap and its potential applications in thin-film solar cells. J. Phys. Chem. Lett. 5, 1289 (2014). 26274486 10.1021/jz500409m

[b12] Okajima M., Endo S., Akahama Y. C. & Narita S. I. Electrical Investigation of Phase Transition in Black Phosphorus under High Pressure. Jpn. J. Appl. Phys. 23, 15 (1984).

[b13] Morita A. Semiconducting black phosphorus. Appl. Phys. A. 39, 227 (1986).

[b14] Luo Z. . Anisotropic in-plane thermal conductivity observed in few-layer black phosphorus. Nat. Commun. 6, 8572 (2014). 26472191 10.1038/ncomms9572 PMC4634212

[b15] Lee S. . Anisotropic in-plane thermal conductivity of black phosphorus nanoribbons at temperatures higher than 100 K. Nat. Commun. 6, 8573 (2014). 26472285 10.1038/ncomms9573 PMC4634207

[b16] Jain A. & McGaughey A. J. H. Strongly anisotropic in-plane thermal transport in single-layer black phosphorene. Sci. Rep. 5, 8501 (2015). 25686917 10.1038/srep08501 PMC4330521

[b17] Cai Y. Q. . Giant Phononic Anisotropy and Unusual Anharmonicity of Phosphorene: Interlayer Coupling and Strain Engineering. Adv. Fun. Mater. 25, 2230 (2015).

[b18] Liu B. L. . Black arsenic-phosphorus: layered anisotropic infrared semiconductors with highly tunable compositions and properties. Adv. Mater. 27, 4423 (2015). 10.1002/adma.201501758 26112061

[b19] Xiang Z. J. . Pressure-induced electronic transition in black phosphorus. Phys. Rev. Lett. 115, 186403 (2015). 26565480 10.1103/PhysRevLett.115.186403

[b20] Akahama Y., Endo S. & Narita S. I. Electrical properties of black phosphorus single crystals. Journal of the Physical Society of Japan 52, 2148 (1983).

[b21] Sugai S. & Shirotani I. Raman and infrared reflection spectroscopy in black phosphorus. Solid Stat Commun. 53, 753 (1985).

[b22] Nilges T., Kersting M. & Pfeifer T. A fast low-pressure transport route to large black phosphorus single crystals. J. Solid State Chem. 181, 1707 (2008).

[b23] Bergmann G. Weak anti-localization-an experimental proof for the destructive interference of rotated spin 1/2. Solid State Comm. 42, 815 (1982).

[b24] Xu G. Z. . Weak antilocalization effect and noncentrosymmetric superconductivity in a topologically nontrivial semimetal LuPdBi. Sci. Rep. 4, 5709 (2014). 25043549 10.1038/srep05709 PMC4104393

[b25] Cha J. J. . Weak antilocalization in Bi_2_(Se_x_Te_1−x_)_3_ nanoribbons and nanoplates. Nano lett. 12, 1107 (2012). 22263839 10.1021/nl300018j

[b26] Chen J. . Gate-voltage control of chemical potential and weak antilocalization in Bi_2_Se_3_. Phys. Rev. Lett. 105, 176602 (2010). 21231064 10.1103/PhysRevLett.105.176602

[b27] Geim A. K. Graphene: status and prospects. Science 324, 1530 (2009). 19541989 10.1126/science.1158877

[b28] Liao Z.-M. . Large magnetoresistance in few layer graphene stacks with current perpendicular to plane geometry. Adv. Mater. 24, 1862 (2012). 22407473 10.1002/adma.201104796

[b29] Pippard A. B. Magnetoresistance in Metals (Cambridge University Press, Cambridge, London, 1989).

[b30] Taskin A. A., Sasaki S., Segawa K. & Ando. Y. Achieving surface quantum oscillations in topological insulator thin films of Bi_2_Se_3_. Adv. Mater. 24, 5581 (2012). 22907834 10.1002/adma.201201827

[b31] Wang K. F., Graf D., Li L. J., Wang L. M. & Petrovic C. Anisotropic giant magnetoresistance in NbSb_2_. Sci. Rep. 4, 7328 (2014). 25476239 10.1038/srep07328 PMC4256591

[b32] Hou Z. P. . High electron mobility and large magnetoresistance in the half-Heusler semimetal LuPtBi. Phys. Rev. B. 92, 235134 (2015).

[b33] Ashcroft N. W. & Mermin N. D. Solid State Physics (Holt, Rinehart and Winston, New York, 1976).

[b34] Abrikosov A. A. Quantum linear magnetoresistance. Europhys. Lett. 49, 789 (2000).

[b35] Parish M. M. & Littlewood P. B. Non-saturating magnetoresistance in heavily disordered semiconductors. Nature 426, 162 (2003). 14614501 10.1038/nature02073

[b36] Xu R. . Large magnetoresistance in non-magnetic silver chalcogenides. Nature 390, 57 (1997).

[b37] Liang T. . Ultrahigh mobility and giant magnetoresistance in the Dirac semimetal Cd_3_As_2_. Nat. Mater. 14, 280 (2015). 25419815 10.1038/nmat4143

[b38] Song J. C. W., Refael G. & Lee P. A. Linear magnetoresistance in metals: Guiding center diffusion in a smooth random potential. Phys. Rev. B. 92, 180204 (2015).

[b39] Narayanan A. . Linear magnetoresistance caused by mobility fluctuations in *n*-doped Cd_3_As_2_. Phys. Rev. Lett. 114, 117201 (2015). 25839304 10.1103/PhysRevLett.114.117201

[b40] Hu J. S. & Rosenbaum T. F. Classical and quantum routes to linear magnetoresistance. Nat. Mater. 7, 697 (2008). 18719705 10.1038/nmat2259

[b41] Friedman A. L. . Quantum linear magnetoresistance in multilayer epitaxial graphene. Nano Lett. 10, 3962 (2010). 20804213 10.1021/nl101797d

[b42] Tang H., Liang D., Qiu R. L. J. & Gao X. P. A. Two-dimensional transport-induced linear magneto-resistance in topological insulator Bi_2_Se_3_ nanoribbons. ACS Nano 5, 7510 (2011). 21793543 10.1021/nn2024607

[b43] Wang Z. H. . Granularity controlled nonsaturating linear magnetoresistance in topological insulator Bi_2_Te_3_ films. Nano Lett. 14, 6510 (2014). 25303407 10.1021/nl503083q

[b44] Soule D. E. Magnetic field dependence of the Hall effect and magnetoresistance in graphite single crystals. Phys. Rev. 112, 698 (1958).

[b45] Thoutam L. R. . Temperature-dependent three-dimensional anisotropy of the magnetoresistance in WTe_2_. Phys. Rev. Lett. 115, 046602 (2015). 26252701 10.1103/PhysRevLett.115.046602

[b46] Han C. Q. . Electronic structure of black phosphorus studied by angle-resolved photoemission spectroscopy. Phys. Rev. B. 90, 085101 (2014).

