# Supplementary material for: Large and Anisotropic Linear Magnetoresistance in Single Crystals of Black Phosphorus Arising From Mobility Fluctuations
Source: Sci Rep. 2016 Mar 31;6:23807. doi: 10.1038/srep23807 (PMC4814878; doi:10.1038/srep23807)
Supplement: Supplementary Information [file srep23807-s1.doc]

Supplementary Material to

**Large and Anisotropic Linear Magnetoresistance in Single Crystals of Black Phosphorus Arising From Mobility Fluctuations**

Zhipeng Hou^1^, Bingchao Yang^2^, Yue Wang^1^, Bei Ding^1^, Xiaoming Zhang^1^, Yuan Yao^1^, Enke Liu^1^, Xuekui Xi^1^, Guangheng Wu^1^, Zhongming Zeng^3^, Zhongyuan Liu^2^ and Wenhong Wang^1,*^

^1^Beijing National Laboratory for Condensed Matter Physics, Institute of Physics, Chinese Academy of Sciences, Beijing 100190, China

^2^State Key Laboratory of Metastable Materials Science and Technology, Yanshan University, Qinghuangdao 066004, China

^3^Key Laboratory of Nanodevices and Applications,Suzhou Institute of Nano-tech and Nano-bionics, Chinese Academy of Sciences, Ruoshui Road 398, Suzhou 215123, China

**(a)**

**(b)**


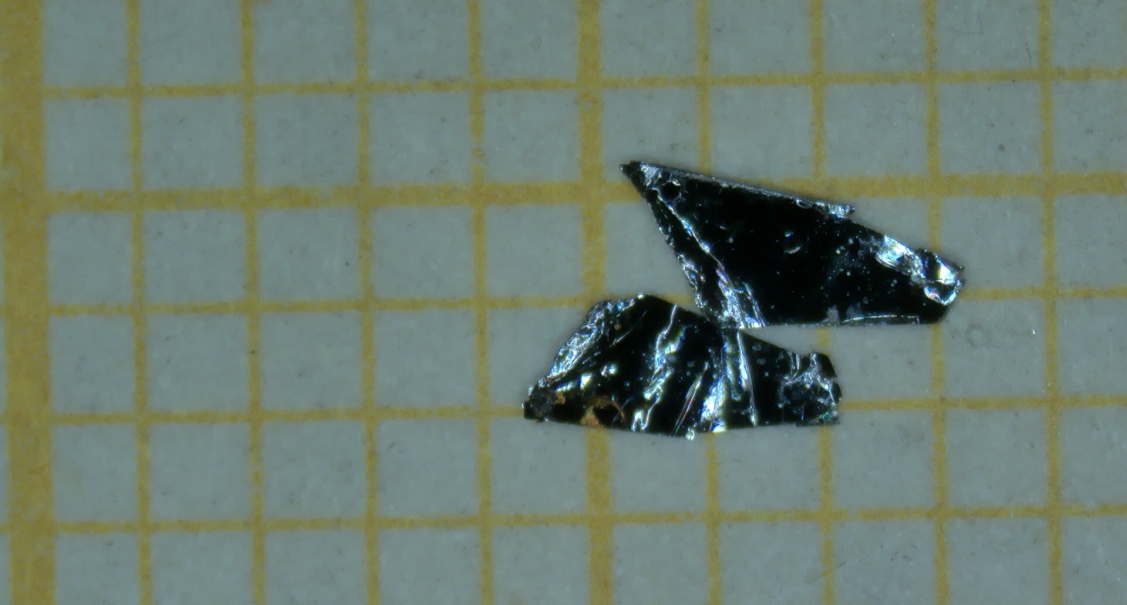
 **(b)**

**(c)**

**(d)**

Figure S1. (a) The typical photograph of the black phosphorus single crystals placed on a millimeter grid with a typical size of about 3.0×1.0×0.1mm^3^. The well-developed surface facing us is established to be (0*l*0) plane by x-ray diffraction, as shown in Figure S1(c). (b) ln(*ρ*_xx_) versus 1/T plot for two pieces of black phosphorus single crystals. The energy gap *E*_g_ at low temperature can be fitted by *ρ*_xx_ (T) = constant×exp(-*E*g/2*κ*_B_*T*), and the corresponding *E*_g_ of the two samples can be established to be 14.6 mev and 15.1mev, which is similar to that of the previously reported bulk BP. (d) Raman spectra collected from cleaved BP. Three major peaks are detected including the out of plane *A*_g_^1^ peak at 362 cm^-1^, the in-plane *B*_2g_ peak at 439 cm^-1^, and the in-plane *A*_g_^2^ peak at 467 cm^-1^.

Figure S2. The field dependence of d*ρ*_xx_/d*B* at different temperatures. It can be seen that the values of d*ρ*_xx_/d*B* starts with a linear increase with the increase of magnetic field from *H* = 0T, indicating that a quadratic MR behavior dominates in the low-field region. With the magnetic field increasing above a crossover field *B*_L_ (The arrow indicates the crossover field), d*ρ*_xx_/d*B* eventually becomes flat suggesting a dominate LMR behavior, though a slight decrease of d*ρ*_xx_/d*B* was detectable at 30K around.

Figure S3. The magnetic field dependence of MR at selected temperatures (The curves measured at 20K, 15K, and 10K are not shown in the main text). At 300K and 200K, it is obvious that MR firstly increases quadratically at the low-field region and then exhibits a linear increase above a crossover field. Below 60K, the quadratic MR regions decreases to so low a field that we did not show them in the figure. Moreover, it is found that the sample exhibits a sublinear MR in the high-field region at 20K and 15K. In the case of 10K, weak-antilocalizaiton (WAL) effect was observed in the low-field region and the high-field region can be fitted with x^2^.

Figure S4. The temperature dependence of MR at various temperatures for sample II. The obvious LMR behavior can also be observed with the magnetic field increases above a crossover point, and we found a maximum MR of 470% at 30K in a magnetic field of 8.5 T which is slightly lower than that of sample I.

Figure S5 The magnetic field dependence of MR in various temperatures for sample II. It is found that the field-dependence MR of sample II is similar to that of the sample I. At 10K, WAL can also be observed though it is not as obvious as that of sample I.

Figure S6 (a), (b) The magnetic field dependence of MR and Hall resistivity at a series of temperatures with the current along *c*-axis. (c), (d) The magnetic field dependence of MR and Hall resistivity at a series of temperatures with the current along *a*-axis. (e), (f), (g) The temperature dependence of MR, hole concentration, and mobility with the current along *a*-axis and *c*-axis.

Figure S7. MR measured at 10K in a maximum field of 10T. No obvious Shubnikov-de Haas (SdH) oscillations were observed.


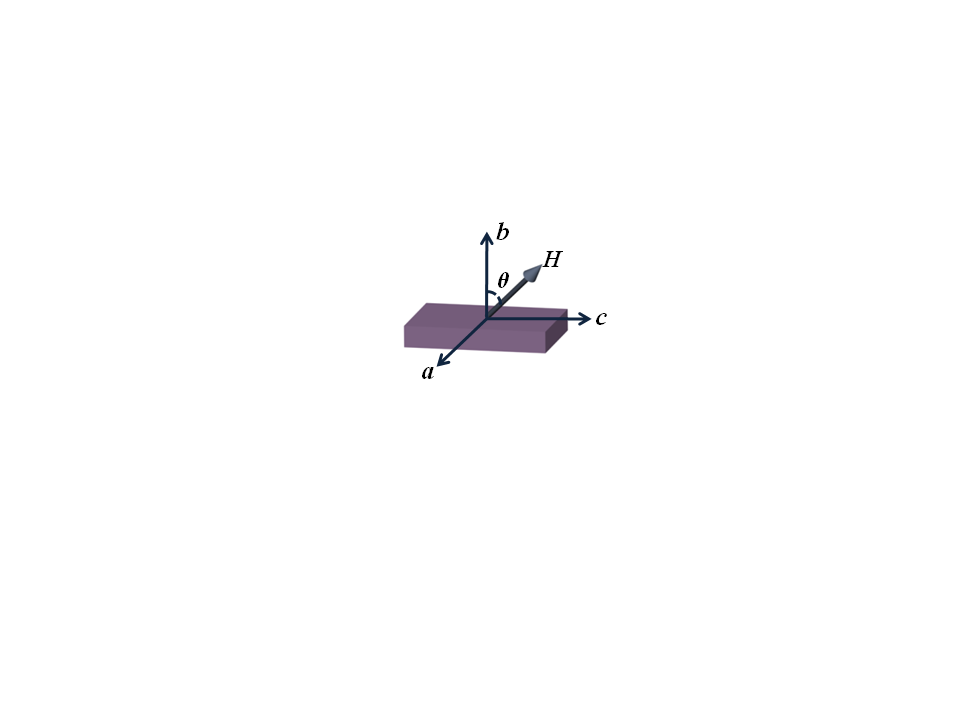


Figure S8. The curves of MR vs the perpendicular component of magnetic field. Inset: the schematic of measurement where *θ* suggests the angle between the magnetic field and the *ac*-plane. *θ* = 90° means the magnetic field is parallel to *ac*-plane.
